# Dynamics of latent HIV under clonal expansion

**DOI:** 10.1371/journal.ppat.1010165

**Published:** 2021-12-20

**Authors:** John M. Murray

**Affiliations:** School of Mathematics and Statistics, UNSW Sydney, Australia; National Cancer Institute, UNITED STATES

## Abstract

The HIV latent reservoir exhibits slow decay on antiretroviral therapy (ART), impacted by homeostatic proliferation and activation. How these processes contribute to the total dynamic while also producing the observed profile of sampled latent clone sizes is unclear. An agent-based model was developed that tracks individual latent clones, incorporating homeostatic proliferation of cells and activation of clones. The model was calibrated to produce observed latent reservoir dynamics as well as observed clonal size profiles. Simulations were compared to previously published latent HIV integration data from 5 adults and 3 children. The model simulations reproduced reservoir dynamics as well as generating residual plasma viremia levels (pVL) consistent with observations on ART. Over 382 Latin Hypercube Sample simulations, the median latent reservoir grew by only 0.3 log_10_ over the 10 years prior to ART initiation, after which time it decreased with a half-life of 15 years, despite number of clones decreasing at a faster rate. Activation produced a maximum size of genetically intact clones of around one million cells. The individual simulation that best reproduced the sampled clone profile, produced a reservoir that decayed with a 13.9 year half-life and where pVL, produced mainly from proliferation, decayed with a half-life of 10.8 years. These slow decay rates were achieved with mean cell life-spans of only 14.2 months, due to expansion of the reservoir through proliferation and activation. Although the reservoir decayed on ART, a number of clones increased in size more than 4,000-fold. While small sampled clones may have expanded through proliferation, the large sizes exclusively arose from activation. Simulations where homeostatic proliferation contributed more to pVL than activation, produced pVL that was less variable over time and exhibited fewer viral blips. While homeostatic proliferation adds to the latent reservoir, activation can both add and remove latent cells. Latent activation can produce large clones, where these may have been seeded much earlier than when first sampled. Elimination of the reservoir is complicated by expanding clones whose dynamic differ considerably to that of the entire reservoir.

## Author summary

The HIV latent reservoir decreases slowly on antiretroviral therapy (ART). However there are cellular processes operating within this reservoir that can expand or contract subpopulations. This means that what is happening at the macro level may not be reflected at the micro level. To investigate this, we analysed published data on HIV latent clone sizes. By constructing an agent model incorporating the processes of cellular activation and proliferation, we were able to show that activation can expand clone sizes significantly even while on ART. Homeostatic proliferation also plays a role in maintaining the reservoir but these clones, though more frequent, are much smaller in size. Our calculations also show that activation and proliferation of the intact latent reservoir can lead to some of these cells becoming virally productive to a level consistent with observed residual viremia during ART. This analysis explains how normal cellular processes restructure the make-up of the latent reservoir and contribute to residual viremia.

## Introduction

Estimates of the latent reservoir half-life under antiretroviral therapy (ART) give a bulk measure of how this collection of cells changes over time [1]. However the degree to which individual cell processes such as homeostatic proliferation and antigen-induced cellular activation, inhibit or accelerate this dynamic is uncertain. So too is the role they play in contributing to residual viremia during ART. Recent data describing frequency distributions of infected cell clonal numbers, provide more detailed data at the individual infected cell level that may help address these questions [2–4]. Infection of a cell results in integration of the viral genome into the host cell’s DNA. By comparing integration positions between infected cells, researchers can determine whether these occurred through different infection events (the integration positions are different), or the cells are clones of a previously infected cell which experienced at least one round of cell division (the integration positions are identical, as are the proviral genomes). These clones can arise through homeostatic proliferation, or from antigen-induced cellular activation, abbreviated simply to activation, where the memory cells remaining after antigen mediated cellular proliferation and clearance, arise from an infected cell that has maintained latency [5]. The relatively high frequencies of some clones in samples of CD4+ T cells, point to very high expansion of these clones to levels that can reach in their millions and that can also contribute to viremia [6]. These clones can also be maintained over many years even in the presence of ART [3]. Although the structure of sampled clonal distributions has been described [7], what these profiles tell about the reservoir’s dynamics at the clonal level have not. A description of clonal dynamics will provide a much more detailed analysis of processes affecting the latent reservoir, and how it may be impacted.

**Fig 1:**
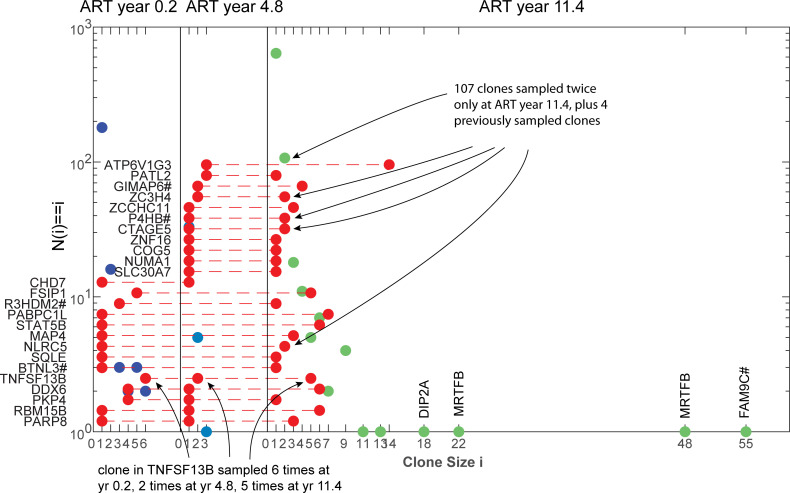
Clone size distributions at years 0.2, 4.8 and 11.4 years after the commencement of ART for Patient 1 of Maldarelli et al. [3]. The red circles denote single clones that are detected at more than one time (each of these denotes a single clone where its position on the *x* axis denotes the size of that clone for each sample, and its *y* position is not counted). The other markers denote the remaining number of clones N(i) of size i that were only detected in the one sample (with frequency on a logarithmic scale). Genes into which the integration is located are shown for clones detected over multiple times and for high clones at year 11.4. # denotes the integration occurred near to that gene.

To investigate the impact these processes have on the latent reservoir, an agent-based model was constructed to track clones (since all integrations will be tracked through the model, the term ‘clone’ will also include singletons (unique integrations)), from the initial infection of an individual at primary HIV infection (PHI) through 10 years prior to the commencement of ART, and followed for a further 11.4 years after ART initiation. Simulating the reservoir both prior to and during ART estimates its impact on the reservoir’s dynamics via its changing clonal structure. The model was calibrated to reproduce the general latent reservoir dynamics as well as clonal data for patient 1 of Maldarelli et al. [3], whose latent reservoir was sampled extensively at three time points, years 0.2, 4.8 and 11.4 after ART initiation (Fig 1, data available from the Retrovirus Integration Database rid.ncifcrf.gov). Additionally simulations were compared to the clone profiles of the four other adults described in Maldarelli et al. [3], as well as the three vertically infected children from Wagner et al. [8].

## Results

**Fig 2:**
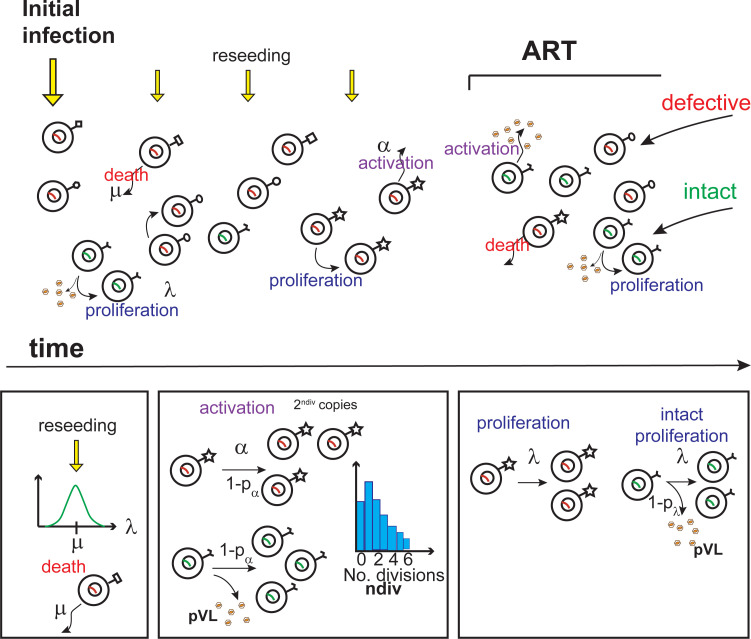
Agent model of latency. Initial infection seeds the latent reservoir to an amount s_0_ cells, and there is continual reseeding prior to ART at each timestep (0.1 years) at the annual rate $s_{0}/\bar{s}$. Both defective (red nucleus) and intact (green nucleus) are seeded, with the latter at 1/100^th^ of the rate of defective latent cells. Cells die at rate μ per cell per year (all times in years), and have the ability to undergo homeostatic proliferation to produce two new clonal cells at rate λ per cell per year, drawn at first integration from a lognormal distribution for each clone with mean *μ* and variance λ_v_. At proliferation, a cell containing genetically intact HIV may instead become sufficiently activated that it progresses to productive infection, with probability 1-p_λ_, and produces virions. Clones are activated at rate α per clone per year but with probability 1-p_α_ produce memory cells from that clone after undergoing *n* rounds of division drawn from a Poisson distribution with mean n_div_. Alternatively, with probability p_α_ activation converts all cells in that clone (up to a maximum of 2^ndiv+mact^ cells) into productively infected cells, in which case intact clones will produce virions and those cells will be eliminated from the reservoir.

The agent model incorporated death of latent cells at rate *μ*, homeostatic cellular proliferation at rate *λ*, and activation of clonal populations at rate *α*. Seeding of the reservoir occurred at PHI but also throughout the period prior to ART initiation. Separate latent populations of cells containing either defective or genetically intact provirus are modelled, the latter population contributing to plasma viremia levels (pVL, HIV RNA copies/mL) when a clone is activated and when proliferation fails to maintain latency and the cell becomes productively infected. By chance, activation may lead to expansion of a clone that remains at high numbers after antigen clearance. A graphical description of the model is shown in Fig 2.

**Fig 3:**
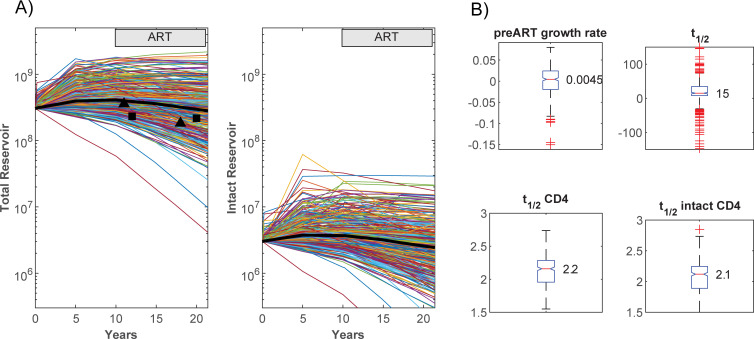
Reservoir dynamics. A) Changes in reservoir size for total and intact components. The black line depicts median values while the markers show integrated HIV DNA copies estimated from two patients in Pinzone et al. [11]. B) Pre-ART reservoir growth rate, reservoir half-life (t_1/2_), half-life of latently infected cells per 10^6^ CD4+T cells (t_1/2_ CD4), and half-life of intact latently infected cells per 10^6^ CD4+T cells (t_1/2_ intact CD4). Estimated pre-ART growth rates were not significantly different from zero. In each plot the box depicts the interquartile range with the median at the notch; red crosses show outliers. The half-lives of total and the intact reservoirs when measured relative to 10^6^ CD4+ T cells were similar at 2.2 and 2.1 years but this was driven by increasing CD4+ T cell numbers over this period (between years 0.2 and 4.8 on ART).

Given uncertainty in parameter values, the model for the intact reservoir component was initially run over 2000 Latin Hypercube samples (LHS) for all parameters, a subset of 382 parameter sets being chosen where the full model reasonably reproduced observed reservoir dynamics (see Materials and Methods). For all 382 parameter sets, the median total reservoir showed little increase in size (0.3 log_10_, Fig 3), over the 10 years prior to ART initiation. Although the latent reservoir can only be directly measured during ART when high numbers of productively infected cells are not contaminating the measurement, some estimate of its dynamics prior to ART has been obtained. Estimates from back-projecting the second phase decay of integrated HIV DNA for a number of individuals where their duration of infection prior to ART were available, indicated latently infected cell numbers increased with duration of untreated infection when assessed relative to 10^6^ CD4+ T cells [9]. That same analysis, when estimated over the whole body, suggests that after the initial infection the latent reservoir is generally stable in number (S1 Fig), as observed here.

Change in reservoir size after commencement of ART is limited. For these simulations, it decreases with a median half-life of 15 years measured in total numbers, while still showing a median decay half-life of 2.2 years when measured relative to 10^6^ CD4+ T cells (and estimated between the 0.2 and 4.8 year time points on ART, Fig 3. Conversion from number of latent cells in the body to per 10^6^ CD4+ T cells was calculated assuming 5.5% of all CD4+ T cells are in peripheral blood [10], distributed through 5×10^6^ μLs and using the patient’s CD4+ T cell count at the time of each sample). The disparity in these changes is due to CD4+ T cells per μL increasing in number from 48 to 185 copies for this patient.

**Fig 4:**
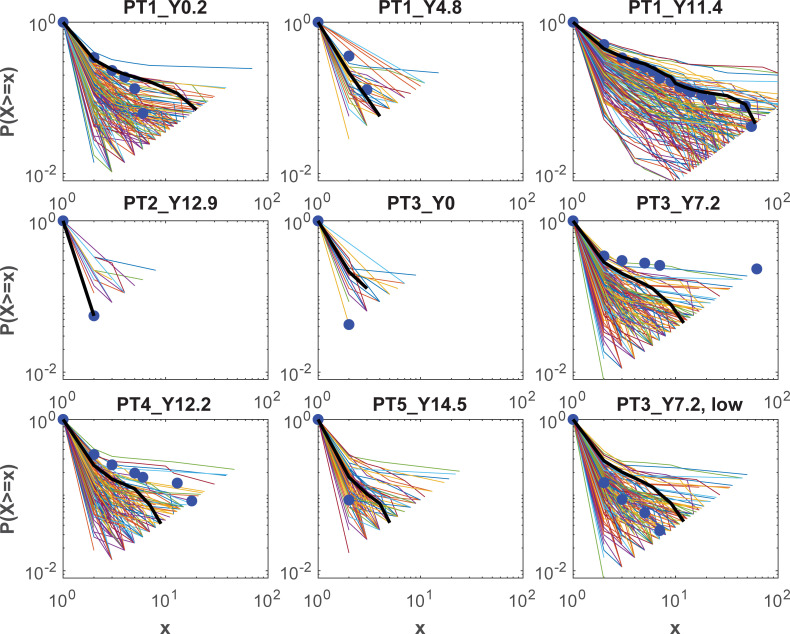
Clone size distributions. Simulated clone distributions versus data on log-log plots for all adult patients, for the simulations achieving clone size ≥10 at the Year 11.4 time point for Patient 1; data as blue circles, individual simulations as coloured lines, the simulation closest to reproducing the year 11.4 data is shown as a black line. The last plot shows simulations and data for Patient 3 at year 7.2 where the highest clone is omitted. For each size value x, the y axis shows the fraction of clones of size x or higher. The general linear trend on these log-log plots is typical of power law distributions.

The model simulations also reasonably reproduced the clone distributions observed for the adult patients [3]. Numerically sampling the same number of latently infected cells as occurred in practice at each time point, and extracting the 145 simulations that exhibited clone sizes ≥10 at year 11.4 for patient 1, produced the distribution of sampled clone sizes shown in Fig 4. The same coloured lines in these plots connect clone size fractions generated within a simulation, displayed as the fraction of clones of that size or higher. The simulation that best reproduced the ART year 11.4 data for Patient 1 is shown as a black line. There was a median of 13 clones that appeared at the first time point (year 0.2) for Patient 1 and were also in the numerical samples at year 11.4 (Fig 4D), the same as the 13 clones cross-sampled for those times in practice (Fig 1). However the simulated high clones at year 11.4 were also mostly detected at the earlier times, which was not the case for this patient. For the only other patient with longitudinal data, Patient 3, there were no clones sampled at both times in practice and a median of 1 clone sampled numerically. It should be noted that both of these patients had complex treatment histories, with Patient 3 undergoing a 10 to 14 day treatment interruption prior to the last sample time, while Patient 1 stopped ART several times between years 4.8 and 11.4, and was diagnosed with lingual squamous cell cancer [3,6]. The treatment interruption at the last time for Patient 3 may have led to the very high clone so that simulations under-estimate the clone distributions. Omitting that size 62 clone produces simulations that encompass the remaining clone sizes (Fig 4, last plot “PT3_Y7.2, low”). Note that all simulations use the same parameter sets so the very different distributions for each patient are due to whatever reservoir changes occur over each time period but are mostly determined by the sample size. How accurately these simulations predict clone size will also have been affected by the time prior to ART and the level of CD4 depletion (unknown period but taken as 10 years for Patient 1 and these simulations (22 CD4+ T cells/μL), 14.8 years for Patient 3 (24 CD4+ T cells/μL), and 6.5 years for Patient 4 (924 CD4+ T cells/μL)).

**Fig 5:**
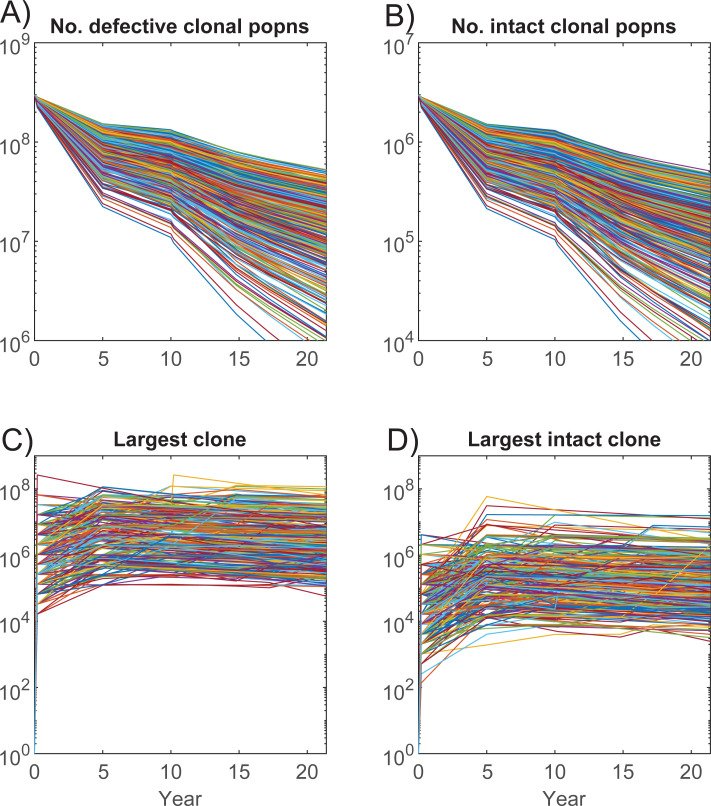
Number and size of clonal populations. Dynamics of number of A) defective and B) intact clonal populations. C), D) Clone size can increase to large levels through activation soon after PHI, but may also expand throughout the course of infection.

Although the reservoir size changes little over time in these simulations (Fig 3), the number of distinct clonal populations decreases for both the defective and intact clones (Fig 5A and 5B). The fast early decay soon after PHI is due to an estimated higher contribution to the reservoir at PHI than through reseeding and the greater chance of a clonal population’s loss when it is of size one, which they all are at first infection. The growth of the reservoir arises from the expansion of clonal populations through proliferation but also activation and survival of those infected clones. Activation gives rise to large clone sizes, while proliferation enhances survival through low level expansion. Here clones can achieve a maximum size of around 10^8^ cells, values similar to estimates of ~10^7^ cells from a sampled intact clone [6]. In these simulations, the lower maximum intact clone size is due to chance and the smaller reservoir from which they are drawn, rather than any lower likelihood of surviving activation.

**Fig 6:**
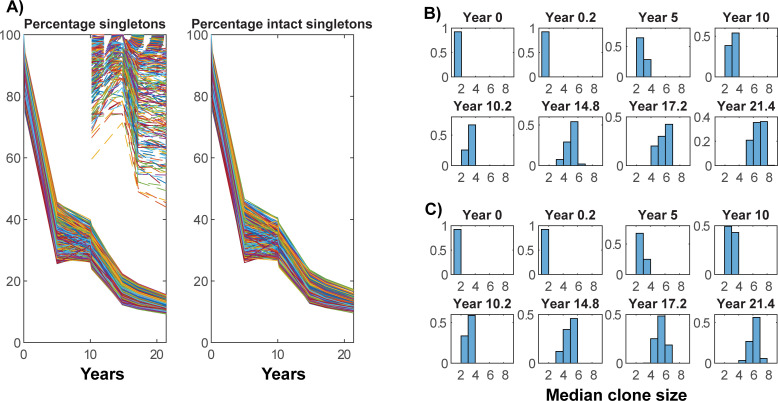
Percentage of singletons and median clone size. Percentage of singletons in the A) total and intact integrations (solid lines) as well as singleton percentage in the numerical samples of sizes 287, 70, 1314, 1314 respectively (dashed lines). Histograms of median clone size for B) defective and C) intact.

The early loss of lower proliferative clones, that are more likely to be singletons, is also shown by their percentage of the total reservoir (Fig 6A). In these simulations most of the reservoir is established at PHI and so the singletons in cells with lower proliferative ability are most likely to be lost soon afterwards while others will expand in number. It is difficult to accurately discern the percentage loss of singletons through sampling, especially when sample sizes differ. However even when sample sizes are the same, as taken in this figure for time points at years 17.2 and 21.4 (1,314 cells), the sampled percentage of singletons does not generally reflect the true dynamic (Fig 6A, dashed lines representing the percentage of singletons in the numerical sample versus solid lines representing their percentage of the whole reservoir), as this depends on the whole profile of clone sizes. The median clone size increases as time progresses, especially when new singletons are not being introduced on ART (Fig 6B and 6C).

**Fig 7:**
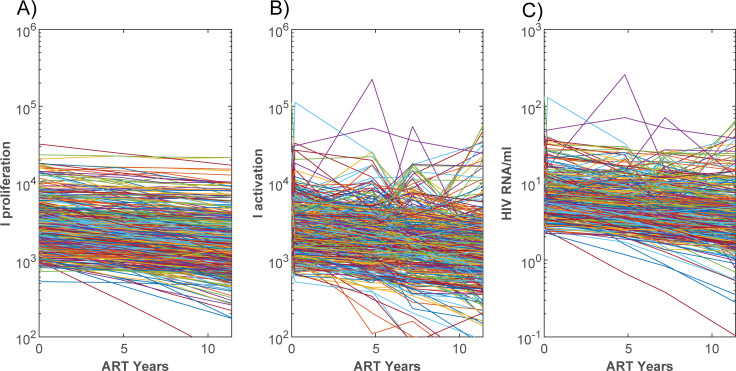
Cells contributing to residual viremia. Number of latent cells in the body that become productively infected due to A) failed homeostatic proliferation, or B) activation. C) Estimated number of HIV RNA copies pr mL during ART originating from these cells.

In this model there are two cellular sources of virions, those that arise from activation of an intact latently infected clone (*I*_*α*_), and those that undergo failed proliferation which instead activates the intact cell (*I*_*λ*_). Scaling these values to per mL of plasma and then through estimates of the steady state virions that they produce (Materials and Methods), gives numbers of HIV RNA copies per mL that are slowly decreasing (Fig 7C). A linear mixed effect model of log(pVL) values during ART determined a yearly decay rate of 0.05126 (95% c.i. [0.0474, 0.0552]) which corresponds to a half-life of 13.5 years (12.6 to 14.6 years), within the range of estimated residual pVL half-lives (95% c.i. of [6.2, 83] years, [12]). Despite their slowly decaying dynamic, some simulations by chance give rise to blips where pVL rise intermittently above the assay detection limit, as observed in some patients [13]. The calculations here are only stored at 4 timepoints after commencing ART so will miss some of the intervening blips that reflect activation of some of the large clones.

### Sensitivity analysis

A sensitivity analysis was conducted to determine the parameters most influential in terms of reservoir and pVL dynamics. Using the original 2,000 LHS parameter sets and the simulations of the intact reservoir component, which parallels the dynamics of the total reservoir (Fig 3), reservoir rates of change prior to and post ART were calculated as well as pVL values at the start of ART and its rate of change during ART. Correlations with the model parameters are listed in S1 Table. Rates of reservoir change, regardless of ART, were most significantly negatively correlated with latent cell death rate *μ*, and positively correlated with the mean number of divisions cells undergo during activation *n*_*div*_. This latter parameter also had the most significant impact leading to higher pVL at the start of ART but also to a greater rate of pVL decay.

### Individual simulations

**Fig 8:**
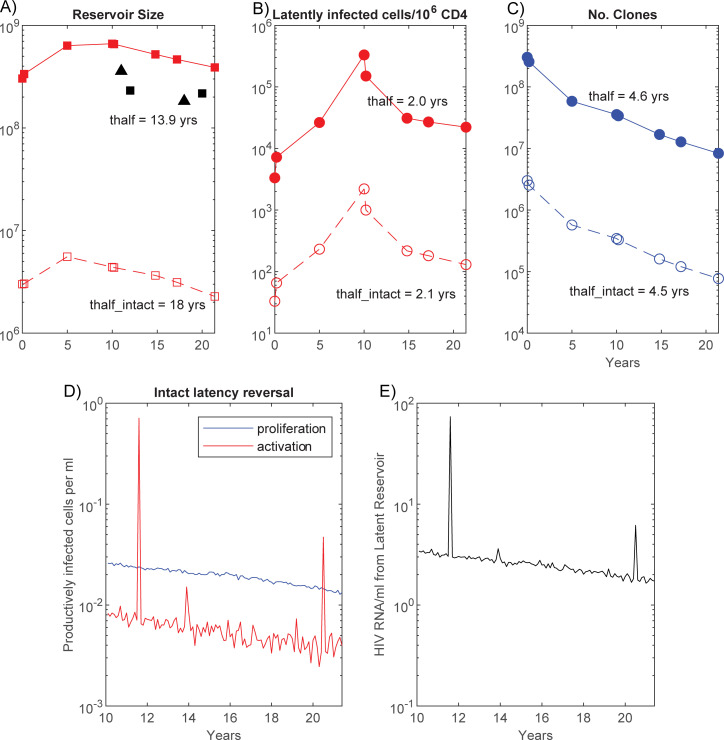
Reservoir and pVL dynamics for the parameter set that best reproduced the year 11.4 ART clone profile. A) The total reservoir is depicted with solid lines and filled markers while the intact component is depicted with dashed lines and open markers, B) the number of total and intact latent cells per 10^6^ CD4+ T cells, C) the number of total and intact distinct clonal populations in the body. The half-lives, calculated between ART years 0.2 and 4.8, are shown for each population. D) the number of cells per mL arising from latent activation or failed proliferation, E) HIV RNA copies per mL originating from failed proliferation and activation. Parameters: μ = 0.8443, λ_v_ = 2.41×10^−5^, α = 0.0044, p_α_ = 0.991, p_λ_ = 0.993, n_div_ = 10, $\bar{s}$ = 42, m_act_ = 7. The mean cell life-span is 1/μ = 1.18 years or 14.2 months.

To provide finer detail on how the clones can change over time, the parameter set was analysed that best reproduced the clonal profile at the 11.4 year timepoint on ART for Patient 1 (Fig 4). As for the multiple simulations, the total reservoir increased over time prior to ART when the larger contribution at PHI is having its greatest impact. The relatively short mean life-span of latent cells in this simulation (14.2 months), resulted in a decaying reservoir on ART, on all measures (Fig 8). The 4.6 year half-life for all clonal populations was shorter than the half-life of the whole reservoir (13.9 years), with the intact reservoir decreasing even more slowly (18 year half-life), likely due to the greater impact on this smaller reservoir of any clones being expanded to high number through activation. The maximum intact clone decreased slightly in size from 4.0×10^5^ cells at 10.2 years to 1.8×10^5^ cells at 21.4 years. Activation during ART ensured that not all clones would decrease however. Of the 18 intact clones of size 10% or higher of the maximum clone size at 21.4 years within this simulation, 5 of these had been small at 10.2 years (≤34 cells), and underwent a mean 7,644-fold increase. Of these 5 expanding clones, 3 had been established at PHI, while one occurred in the year prior to ART initiation. The dynamics of the total reservoir will not be replicated by all individual clonal populations, and activation plays both roles of increasing some, while removing others to contribute to residual pVL (Fig 8).

The shift over time to higher proliferating clones, is due to their greater likelihood to survive to expansion through either proliferation or activation (S2 Fig), as has been described by others [14], so that higher levels of detection of integrations in oncogenic sites are less likely due to HIV preferentially integrating in these locations [8].

For this calculation, residual viremia decayed over time with a half-life of 10.8 years, mostly tracking the higher contribution from proliferation than activation in this instance (Fig 8D and 8E). As antigen-induced cellular activation is more likely to convert a large number of latent cells within an intact clone to productive infection, virion numbers produced by activation may vary dramatically over time, and in this simulation gave rise to intermittent viral blips of a clonal origin, as has been observed in practice [15]. On the other hand, proliferation will act at an average rate over the entire reservoir and hence will contribute smoother changes in pVL. Individuals for which activation is higher will have much more varied pVL profiles and experience more frequent blips.

**Fig 9:**
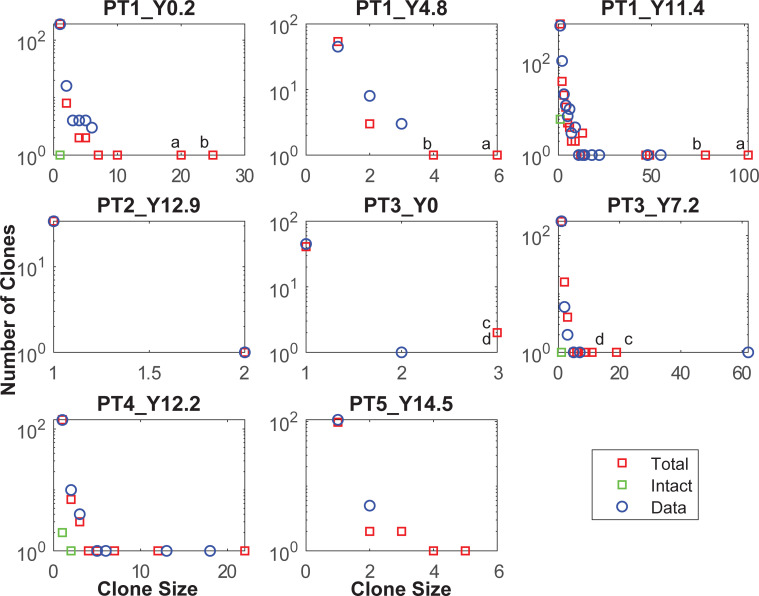
Clone size distributions for all adult patients. The simulated total (red squares) and intact (green squares) clone sizes for the same sampled number of latently infected cells as the data (blue circles), for all adult patients described by Maldarelli et al., [3]. All simulations were performed with the same parameter set as described in Fig 8. The two largest simulated clones, size 102 (a) and 79 (b), for patient 1 at Y11.4 of ART are also shown at earlier times; similarly the two largest simulated clones, sizes 19 (c) and 9 (d), for patient 3 at Y7.2 of ART.

The clone size distributions for the adult patients were also reasonably reproduced, despite the simulations using a single parameter set, the stochasticity of the computations, and all assuming a 10 year duration prior to ART initiation (Fig 9). However the largest simulated clones for Patients 1 and 3 at the last sampled times, were also contained in previous samples, in contrast to the data. The larger simulated samples included intact clones, but these were much smaller than the defective clones.

**Fig 10:**
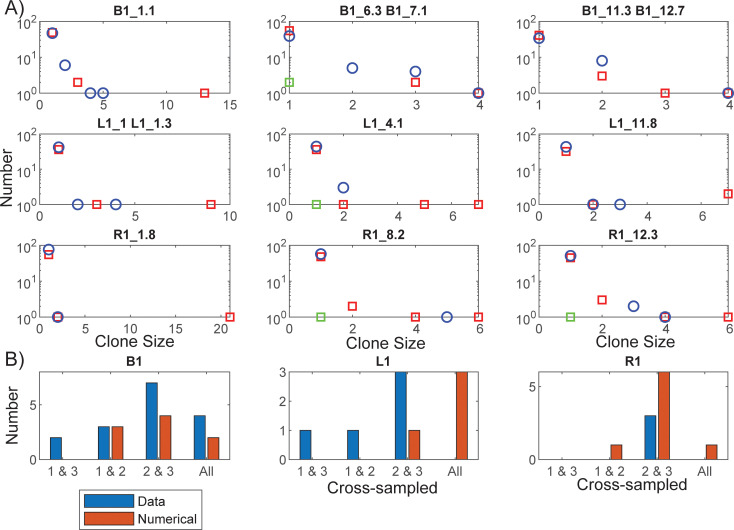
Clone size distributions for all child patients. A) The simulated total (red squares) and intact (green squares) clone sizes for the same sampled number of latently infected cells as the data (blue circles), for all children data described by Wagner et al. [8]. All simulations were performed with the same parameter set as described in Fig 8. The only modification to the simulations were starting ART at 1 year after infection and assuming a 10-fold lower starting reservoir, with proportionately lower reseeding. B) Number of clones cross-sampled at the various times for the data (blue bars) and the simulations (red bars).

The other data to which model simulations were compared were for young children vertically infected with HIV [8]. These children had much shorter periods prior to ART initiation, in these simulations taken to be one year. The number of CD27+ memory CD4+ T cells significantly increase from birth to adulthood at the rate of 0.7 per year [16], so that young children will have a lower potential latent reservoir than an adult. Assuming a 10-fold lower starting latent reservoir, and a proportionately lower reseeding rate, with ART commenced one year after infection, leads to clonal distributions for these children shown in Fig 10A. These reasonably match the data for their clone distributions, as well as the number of times clones were recorded at multiple time points Fig 10B, although the simulations tended to produce larger sized clones than sampled.

### The relative contributions of proliferation and activation

To further dissect the relative contributions to the latent reservoir of each process that contributes to clonal expansion, a model tracking clonal size dynamics was developed where expanding clone numbers only occurred through proliferation–activation was assumed only to remove clonal populations (S1 Text). The dynamics of the number of clones of size *i*, when only considering expansion through proliferation at a fixed rate *λ*, is approximately described by a power law $D_{i}\approx \bar{D}\;z^{i-1},i=1,2,\ldots$. At PHI the value of *z* is small since all clones are size 1, but as infection continues, some will expand under proliferation *λ* in spite of loss through death *μ* and activation *α*, so that some clones will grow in size and the value of *z* increases over time to the smaller of the values *λ/μ* or 1, according to the equation

$$\frac{dz}{dt}=\mu (z-1)\left(z-\frac{\lambda }{\mu }\right)$$

**Fig 11:**
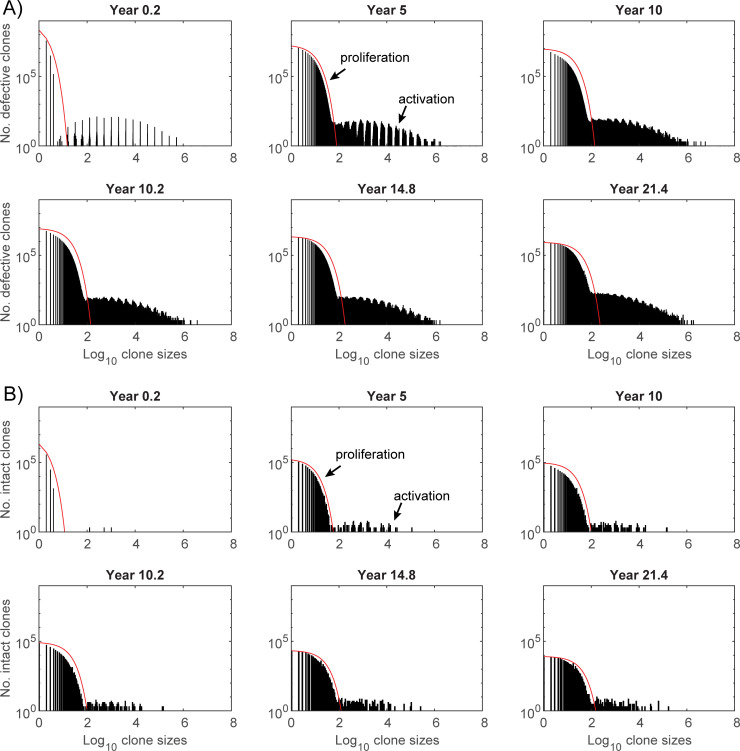
Histograms of clonal sizes for the individual simulation with parameters as described in Fig 8. Histograms of A) defective and B) intact clone sizes. First row in each plot shows times prior to ART, while second row shows times during ART. Shown as red lines are clone size estimates from the proliferation model using the number of clones of size *i* given by $D_{i}=\bar{D}\;z^{i-1}$ where $\frac{dz}{dt}=\mu (z-1)\left(z-\frac{\lambda }{\mu }\right)$ with $\frac{\lambda }{\mu }=0.99$ and $z(0.2)=0.01\frac{\lambda }{\mu }$. Under these calculations, the largest intact clone size due solely to proliferation at the last time point is size 154 (*i* such that $\bar{D}\;z^{i-1}\approx 1$).

This dynamic of proliferating clones provides a good fit to the agent based simulations for the component driven by proliferation (Fig 11, comparison with calculations for children are displayed in S3 Fig.). Under these calculations, the largest defective clone size driven solely be proliferation will be size 232 at the last time point. The probability a clone of this size would be sampled more than once at that time from the estimated 389 million latently infected cells is *p* = 3.1×10^−7^. Hence the clones sampled multiple times will almost certainly have arisen through activation. Proliferation can expand these clones, but activation and survival from that process will be the driver in generating these large clones.

The mean clone size for the proliferative component is given by

$$Mean\;clone\;size=\frac{\sum_{i}iD_{i}}{\sum_{i}D_{i}}\approx \frac{1}{1-z}$$

and since *z* increases over time, and especially when new infections are restricted on ART, the mean clone size will also increase. Under this calculation the mean proliferating clone size is 17.5 at the last time point. The mean size of all clones in the agent based simulation is much higher at 46.7 since it will include the very high sizes arising from activation as well. On the other hand, the median clone size is only 7 mostly determined by the larger number of clones that have expanded by proliferation (8,315,740 of size 232 or less) than activation (19,160).

## Discussion

These simulations incorporate the chance events establishing and maintaining the latent reservoir. For certain parameter values they reproduce not only the gross dynamics of the reservoir, but also the more clone-specific elements as observed through longitudinal sampling. The reservoir dynamics were most affected by the average latent cell death rate *μ*, so that a higher death rate leads to less reservoir growth prior to ART and greater decay during ART, but where this can be offset by a lower activation rate *α*, and a larger number of divisions a surviving clone has undergone *n*_*div*_. If residual pVL is to change little during ART, then these two aspects related to activation will be low (S1 Table).

For the parameter subsets that reasonably reproduced the limited available longitudinal data [11] (Fig 3), the number of clonal populations within the reservoir always decreased with time, despite the median total number of latent cells showing little change. Both proliferation and expansion through activation compensated the loss in clone numbers. The large clone sizes within the reservoir and observed via sampling, were produced by activation, since this latter process generated the largest clone sizes while proliferation on its own produced much smaller clones (Fig 11). That antigen-induced cellular activation is mainly responsible for very large clones, is consistent with higher clonality in CMV versus Gag-responding CD4+ T cells [17]. The relativities of proliferation and activation also impacted on variability of residual pVL and the occurrence of viral blips (Fig 8).

The parameter set that best reproduced the simulated clone profile for the patient 1 data of Maldarelli et al. [3], exhibited a relatively short latent cell lifespan of 14.2 months. Despite this, activation and proliferation were able to partially compensate for reservoir loss and resulted in decay of the total reservoir with a 13.9 year half-life on ART (Fig 8). The model only includes a single latent cell phenotype, except for allowing variance of proliferation around the mean, so the best fit average lifespans of 14.2 months, much shorter than 20 year estimates [18], may be a composite of resting cells and those with a more activated phenotype. Transition between HLA-DR negative and positive [19,20], may contribute to this lower estimated average cell life-span while incorporating the processes of proliferation. The 18% annual increase in infected HLA-DR memory CD4+ T cells during ART [20], would impact the continued reservoir decay, to an eventual slower reservoir loss or even rise. This evolution in proliferation of the reservoir would also impact on its decay rate relative to a set number of CD4+ T cells or their resting component.

Homeostatic proliferation by itself will not produce the large sampled clone sizes. Under these calculations, it is responsible for the expansion and survival of the majority of clones but that differ only marginally in size (Fig 11), and when sampled will contribute almost exclusively to singletons. The large sampled clone sizes have been generated through activation, regardless of ART. The largest clone sizes sampled at year 11.4 of ART for patient 1 had not been previously sampled which would indicate that they had been expanded in number some time during ART. This patient however had stopped ART for a period prior to this time, during which the integration may have occurred. Proliferation by itself can produce high clone sizes if their proliferative capacity is very high, but then these clones would continue to expand relative to lower proliferative clones, so that they would completely dominate samples obtained at later times. Most multiply sampled clones for this patient that also occurred at all sample times (Fig 1), were relatively stable in proportion indicating that they were not driven by continuing high proliferation.

Activation, while responsible for the removal of most clonal populations, also plays a role in off-setting some of the macro loss of the latent reservoir through large expansions of other clones, which may have even been established at PHI and stayed low in number till much later. Time to latent eradication is under-estimated when based on the entire reservoir since simulations exhibiting a 2.1 year intact reservoir half-life relative to 10^6^ CD4+ T cells, also contained individual clones that expanded markedly, and was not indicative of the 18 year half life of the intact reservoir in toto (Fig 8). This activation rate produces an average 27 cells per day from the intact reservoir at the final time, considerably higher than previous estimates of 4 cells/day or lower, based on time to viral rebound after ART cessation [21]. The model does not include target cells so cannot simulate time to rebound, and therefore it is uncertain how it will be impacted by this level of activation and the slower decay of the reservoir than previous 44 month estimates [1]. Nevertheless, time to viral rebound can be affected by many factors, not the least being the nonlinearity of viral dynamics [22,23]. That activation occurs continually in these simulations while not always leading to total reservoir loss during ART (Fig 3), indicates that latency reversing agents would need to be potent to add significantly to reservoir loss.

These simulations indicate that a slowly decaying latent reservoir need not indicate cells within the reservoir are long-lived. In the simulation shown in Fig 8, a reservoir decaying with a 13.9 year half-life was established with cells described by an average 14.2 month half-life. Even with a greatly reduced annual reseeding that is 1/42^nd^ of that provided at PHI, proliferation and activation were sufficient to maintain the reservoir and produce the observed clonal profiles. As a result of this low reseeding value, the vast majority of the reservoir was established at PHI, 79% within the first year of infection compared to only 2.4% in the year prior to ART (S2 Fig). This is markedly different to results suggesting the majority of the reservoir is established in the year before ART initiation [24–26], and even to findings where it is established more uniformly during untreated infection [15]. To achieve either of these results requires a higher rate of reseeding and a higher clearance rate if the reservoir is to be relatively stable in number after PHI. The second best-fit simulation had approximately double the rate of reseeding and did contribute more to the reservoir from the last year before ART (9.7% versus 59% within the first year of infection, S4 Fig), but also produced a larger increase in reservoir size, despite latent cells exhibiting an 11.9 month mean life-span. Although the initial 2,000 simulations covered a wide range of parameter values they cannot be exhaustive, and there may be some choices that better reflect the reservoir and clonal dynamics and also the time contribution to the reservoir. For that to happen would require a balance where the higher reseeding is offset by even greater clearance. In these simulations proliferation is assumed roughly equal to the death rate, so a greater loss would then need an average proliferation rate lower than the death rate, leading to a faster overall reservoir decay at rate μ+α-λ (S1 Text). However these rates may not be independent of ART status, as was assumed here. Superinfection of latent cells prior to ART and their faster clearance than during treatment can also achieve a preferential removal of latent cells established earlier in infection, with a slower dynamic during ART [27].

Although the model and calculations have been constructed to represent as many agents in the model as cells in the latent reservoir, they include a number of simplifications that will affect their ability to truly duplicate its behaviour. All latently infected cells are assumed to have the same approximate lifespan whereas the reservoir is composed of cells with different activated and resting phenotypes [19,20,28]. No differences were assumed in the ability of intact or defective clones to survive activation to produce new memory cells from these clones. The memory cells established after clearance of their antigen can be very large, reaching sizes in excess of one million (Fig 5), which would correspond to more than 20 cell doublings from a single infected cell. This is much larger than CD4+T cell expansions that are expected to be of the order of 1,000 fold of which more than 95% would die after antigen clearance [29]. Nevertheless expansions of this magnitude have been observed for even intact clones [6]. High clonal sizes sampled from the reservoir also point on a proportional basis to expansion through activation and subsequent survival of cell doublings of this magnitude (Fig 1) [3].

In conclusion, these agent-based model simulations of the establishment and maintenance of the latent reservoir produce dynamics and clonal size profiles consistent with observations. They also produce pVL at the expected low level via activation of intact clones or proliferation. These calculations point to a reservoir where the size and dynamics of the entire reservoir is not necessarily reflected in the behaviour of individual clones, which can expand to a high degree even while the reservoir is decaying on ART. This will impact prospects for elimination.

## Materials and methods

The typical ordinary differential equation model cannot suitably incorporate stochastic events such as when a particular clone will proliferate or die, but instead describe systems where parameters and the resulting simulations represent average behaviour. On the other hand, agent-based models can assign different behaviour to each agent, so that these models are more suitable for describing the generation and dynamics of clones that may have different proliferative abilities or where an event such as activation may or may not happen. The pre- and post-ART times over which the agent model was run, were chosen to reproduce the data for Patient 1 of Maldarelli et al. [3], who commenced ART at a CD4+ T cell count of 22 cells/μL (indicating a long history of untreated infection), and whose infected CD4+ T cells were then sampled at years 0.2, 4.8 and 11.4 on ART. Given the low CD4+ T cell count at ART initiation, time to ART was set at 10 years to encompass the median time of 7.7 years to AIDS for an individual of this age [30].

As well as establishing integration events at PHI [31], studies of perinatally infected children undergoing periods of one or two-drug regimens prior to enrollment on ART, show the establishment of latent viral genomes with mutations resistant to the non-suppressive drugs [32]. The slow establishment of drug resistant mutations for non-suppressive regimens was also observed in adults who had initially been enrolled on a lamivudine pre-HAART regimen and who developed the resistant 184V mutation [33]. Hence, in the model, latent cells, both intact and defective, are assumed established at initial infection but with additional reseeding over the period prior to ART initiation from new infection events where the cells revert to latency (Fig 2). The model stored the establishment time of each clone. Here cells containing genetically intact virus are assumed to be capable of producing virions when reverting from latent to productive infection. Since ART essentially halts productive infection [34–36], no reseeding is assumed once ART is initiated so that the reservoir will decay dependent on the levels of activation, death and proliferation.

Given that incorporation of the viral genome into each cell can occur at different positions within the host cell genome, including at oncogenic sites which may lead to higher rates of proliferation [8], or that the cells themselves may inherently differ in proliferative ability relative to death [14], it is assumed that each latent cell is established with a proliferation rate *λ*_*i*_ that is randomly chosen from a lognormal distribution with mean *μ* given by the average latent cell death rate (all time is in years, Fig 2). If a cell within a clone is chosen to divide (from a binomial distribution at rate *λ*_*i*_) then that cell will be replaced by two identical daughter cells with the same proliferative rate. Homeostatic proliferation of a cell, which increases the reservoir by one, and death, which decreases the reservoir by one, are chosen to be approximately equal so that in the absence of HIV infection CD4+ T cell numbers are approximately constant. In these calculations, cells chosen to die are removed prior to calculations of proliferation. By chance (probability *1-p*_*λ*_), cells containing genetically intact virus may instead up-regulate viral protein synthesis upon proliferation to the point where they become productively infected, thereby producing virions and with that cell being cleared from the reservoir (“failed homeostatic proliferation”). Activation is assumed to occur over the entire clone (up to a maximum of *2*^*ndiv+mact*^ cells within the clone), since they will all recognise the same antigen, at the rate of *α* clonal populations per year. The maximum clearance amount of large clones on activation allows observations of clonal pVL over extended periods [3,6]; also when clones can reach sizes of 9 million cells [6], it is unlikely that any single event would be able to activate the entire clone. Upon activation of a clone, expansion of these memory cells will produce a subset that remains after activation to act as further memory cells for future occurrences of that antigen. By chance (probability *1-p*_*α*_) replacement memory cells will be produced from a cell containing the viral genome which will undergo *n*_*div*_ rounds of division (Poisson distributed) producing 2^*ndiv*^ clonal copies. If this occurs for a clone containing intact virus, then the cell will instead, with probability *p*_*α*_, become productively infected, giving rise to virion production.

Simulations of the processes included in the model and described in the previous paragraph, are used to determine how they can produce the observed rate of change of the latent reservoir on ART as well as the distribution of clone sizes found in the longitudinal samples of this patient. However they may also explain the source of residual viremia during ART. Under these assumptions, there are two cellular sources of virions, those that arise from activation of an intact latently infected clonal population (*I*_*α*_), and those that undergo failed proliferation which instead activates the intact cell (*I*_*λ*_). Virion production by these cells follows the dynamic $\frac{dV}{dt}=N(I_{\alpha }+I_{\lambda })-cV$, where *N* denotes the number of virions exported by a productively infected cell per day and *c* is the daily loss rate of virions; since the clearance rate of virions is much faster than the loss rate of productively infected cells, this differential equation is in a quasi-steady state so that $V\approx \frac{N}{c}(I_{\alpha }+I_{\lambda })$. This can be translated into HIV RNA copies per ml assuming virions are distributed in the same proportion as percentage of CD4+ T cells in a mL of peripheral blood, and where 5.5% of all CD4+ T cells are in peripheral blood [10] and distributed through 5,000 mLs. With 2 HIV RNA copies per virion, *c* = 23 per day [37], and *N* = 1150 (in the range of 1,000 to 50,000 virions per day [38]), this gives HIV RNA copies/mL $\approx 2\times \frac{N}{23}\times \frac{5.5}{100}\times \frac{1}{5000}\times (I_{\alpha }+I_{\lambda })=0.0011(I_{\alpha }+I_{\lambda })$.

The reservoir consists of cells containing either defective or intact virus. Here cells with intact virus are assumed capable of producing virions once activated, or via failed homeostatic proliferation. Depending on how it is measured, the intact latent reservoir is estimated to be in the range of 10^5^ to 10^7^ cells [39–41]. These measurements depend on how ‘intact’ is determined, for example, through the analysis of the packaging and *env* regions of the viral genome with the intact proviral DNA assay (IPDA), with the quadruplex PCR with four probes (Q4PCR) assay, or via a quantitative viral outgrowth assay (QVOA) [42,43]. It is also affected by whether it is only measured in resting CD4+ T cells [44], or whether cells with less of a resting phenotype are included, such as those expressing HLA-DR [20], or indeed all of CD4+ T cells [3]. As a consequence the proportion of the reservoir that is intact also varies, with intact relative to total estimates of 11.7% [45], 2.4% [2], or down to 1,000 fold lower [43]. Therefore in these calculations, the number of intact latent cells seeded initially was assumed to be 3×10^6^ cells, with the defective reservoir 100-fold higher. Reseeding also occurred with this ratio of intact versus defective.

Given uncertainty in parameter values, the model for the intact reservoir component was initially run over 2000 Latin Hypercube samples (LHS) for all parameters, with the uninfected CD4+ T cell death rate over a range that ran from an average lifespan of 8 months to 20 years (*μ*∈[0.05, 1.5]), values that encompass the estimated 20 year average time to death for T cells, and close to the 22 week average time to division for memory CD4+ T cells. However simulations allowing up to a 20 year average death rate failed to reproduce a relatively stable latent reservoir prior to ART and at the same time generate large clone sizes observed at the last time for Patient 1. Hence this parameter range was restricted to *μ*∈[0.3, 1.5]. The 100-fold lower intact reservoir calculations were considerably faster than the full model, and when rescaled to the size of the total reservoir produced estimates for their dynamics. A subset of 413 parameter sets were extracted that produced “reasonable levels” in terms of reservoir size and pVL levels. Pinzone et al. [11] had estimated levels of integrated HIV DNA per μL of blood for 2 individuals at time points during ART (Subjects 1 and 2, Supp Table 3, Pinzone et al.). Simulations were extracted that maintained reservoir levels at years 0.2 and 7.2 on ART, within 0.5 logs of two data points: log10(ReservoirSize(10.2)perμL)≤log10(3.58)+0.5&log10(ReservoirSize(17.2)perμL)≥log10(2.17)−0.5. Additionally parameter sets were restricted so that simulated pVL values at the start of ART were no more than 10 copies per mL [12,46]. The 413 parameter sets were run on the full model and the 382 simulations that completed within 24 hours clocktime (each running on 12 CPU nodes and a maximum of 64GB of memory) were retained for analysis.

The 2,000 LHS parameter sets were obtained over bounds on the parameters: $\mu \in [0.3,1.5],\;\lambda_{s}\in [0.005,0.02],\;\alpha_{s}\in [0.005,0.05],\;p_{\alpha }\in [0.96,0.995],\;p_{\lambda }\in [0.96,0.995],\;n_{div}\in [6,\ldots ,10],\;\bar{s}\in [14,\ldots ,50],\;m_{act}\in [1,\ldots ,14]$, where for each set $\lambda_{v}=\mu \lambda_{s}^{2},\;\alpha =\mu \alpha_{s}$ to maintain relativities with proliferation. Sampling for the probabilities were determined after logit transformations.

Tracking the initial 3.03×10^8^ latent cells and subsequent expansions/additions/deletions was computationally expensive. Hence comparisons with patient data was estimated from simulations evaluated at the closest times of 0.2, 4.8, 7.2 and 11.4 years on ART, the sample times for Patient 1 and the last time for Patient 3 of Maldarelli et al. [3]. For example comparisons to the last sample for R1 at year 12.3 was determined by numerically sampling the reservoir at year 11.4 on ART.

All calculations were performed with Matlab R2021a, Mathworks Inc., Natick MA, USA.

## Supporting information

S1 Fig Latent reservoir dynamics prior to ART. (DOCX)

S2 Fig Changes in the proliferative profile and the times of establishment of each clone in the first individual simulation. (DOCX)

S3 Fig Histograms of clonal sizes for the child simulation. (DOCX)

S4 Fig Simulations with higher levels of reseeding. (DOCX)

S1 Table Sensitivity analysis. (DOCX)

S1 Text Clonal expansion under proliferation. (DOCX)

## References

[1] Finzi D., Blankson J., Siliciano J.D., Margolick J.B., Chadwick K., Pierson T., et al., Latent infection of CD4+ T cells provides a mechanism for lifelong persistence of HIV-1, even in patients on effective combination therapy. Nature Medicine, 1999. 5(5): p. 512–7. doi: 10.1038/8394 10229227

[2] Bruner K.M., Wang Z., Simonetti F.R., Bender A.M., Kwon K.J., Sengupta S., et al., A quantitative approach for measuring the reservoir of latent HIV-1 proviruses. Nature, 2019. doi: 10.1038/s41586-019-0898-8 30700913 PMC6447073

[3] Maldarelli F., Wu X., Su L., Simonetti F.R., Shao W., Hill S., et al., Specific HIV integration sites are linked to clonal expansion and persistence of infected cells. Science, 2014. 345(6193): p. 179–183. doi: 10.1126/science.1254194 24968937 PMC4262401

[4] Wang Z., Gurule E.E., Brennan T.P., Gerold J.M., Kwon K.J., Hosmane N.N., et al., Expanded cellular clones carrying replication-competent HIV-1 persist, wax, and wane. Proceedings of the National Academy of Sciences, 2018. 115(11): p. E2575–E2584. 10.1073/pnas.1720665115 PMC5856552 29483265

[5] Musick A., Spindler J., Boritz E., Pérez L., Crespo-Vélez D., Patro S.C., et al., HIV Infected T Cells Can Proliferate in vivo Without Inducing Expression of the Integrated Provirus. Front Microbiol, 2019. 10: p. 2204. doi: 10.3389/fmicb.2019.02204 31632364 PMC6781911

[6] Simonetti F.R., Sobolewski M.D., Fyne E., Shao W., Spindler J., Hattori J., et al., Clonally expanded CD4^+^ T cells can produce infectious HIV-1 in vivo. 2016. 113(7): p. 1883–1888. doi: 10.1073/pnas.1522675113 26858442 PMC4763755

[7] Reeves D.B., Duke E.R., Wagner T.A., Palmer S.E., Spivak A.M., and Schiffer J.T., A majority of HIV persistence during antiretroviral therapy is due to infected cell proliferation. Nature Communications, 2018. 9(1): p. 4811. doi: 10.1038/s41467-018-06843-5 30446650 PMC6240116

[8] Wagner T.A., McLaughlin S., Garg K., Cheung C.Y.K., Larsen B.B., Styrchak S., et al., Proliferation of cells with HIV integrated into cancer genes contributes to persistent infection. Science, 2014. 345(6196): p. 570–573. doi: 10.1126/science.1256304 25011556 PMC4230336

[9] Murray J.M., McBride K., Boesecke C., Bailey M., Amin J., Suzuki K., et al., Integrated HIV DNA accumulates prior to treatment while episomal HIV DNA records ongoing transmission afterwards. AIDS, 2012. 26(5): p. 543–550. doi: 10.1097/QAD.0b013e328350fb3c 22410637

[10] Savkovic B., Macpherson J.L., Zaunders J., Kelleher A.D., Knop A.E., Pond S., et al., T-lymphocyte perturbation following large-scale apheresis and hematopoietic stem cell transplantation in HIV-infected individuals. Clinical Immunology, 2012. 144(2): p. 159–171. doi: 10.1016/j.clim.2012.06.004 22772031

[11] Pinzone M.R., VanBelzen D.J., Weissman S., Bertuccio M.P., Cannon L., Venanzi-Rullo E., et al., Longitudinal HIV sequencing reveals reservoir expression leading to decay which is obscured by clonal expansion. Nature Communications, 2019. 10(1): p. 728. doi: 10.1038/s41467-019-08431-7 30760706 PMC6374386

[12] Riddler S.A., f.t.A.A.P. Team, Aga E., f.t.A.A.P. Team, Bosch R.J., f.t.A.A.P. Team, et al., Continued Slow Decay of the Residual Plasma Viremia Level in HIV-1–Infected Adults Receiving Long-term Antiretroviral Therapy. The Journal of Infectious Diseases, 2015. 213(4): p. 556–560. doi: 10.1093/infdis/jiv433 26333941 PMC4721905

[13] Di Mascio M., Markowitz M., Louie M., Hogan C., Hurley A., Chung C., et al., Viral blip dynamics during highly active antiretroviral therapy. Journal of Virology, 2003. 77(22): p. 12165–72. doi: 10.1128/jvi.77.22.12165-12172.2003 14581553 PMC253757

[14] Coffin J.M., Bale M.J., Wells D., Guo S., Luke B., Zerbato J.M., et al., Integration in oncogenes plays only a minor role in determining the in vivo distribution of HIV integration sites before or during suppressive antiretroviral therapy. PLoS Pathog, 2021. 17(4): p. e1009141. doi: 10.1371/journal.ppat.1009141 33826675 PMC8055010

[15] Jones B.R., Kinloch N.N., Horacsek J., Ganase B., Harris M., Harrigan P.R., et al., Phylogenetic approach to recover integration dates of latent HIV sequences within-host. Proceedings of the National Academy of Sciences, 2018. 115(38): p. E8958–E8967. doi: 10.1073/pnas.1802028115 30185556 PMC6156657

[16] Hazenberg M.D., Otto S.A., van Rossum A.M.C., Scherpbier H.J., de Groot R., Kuijpers T.W., et al., Establishment of the CD4+ T-cell pool in healthy children and untreated children infected with HIV-1. Blood, 2004. 104(12): p. 3513–3519. doi: 10.1182/blood-2004-03-0805 15297312

[17] Simonetti F.R., Zhang H., Soroosh G.P., Duan J., Rhodehouse K., Hill A.L., et al., Antigen-driven clonal selection shapes the persistence of HIV-1-infected CD4+ T cells in vivo. J Clin Invest, 2021. 131(3). doi: 10.1172/JCI145254 33301425 PMC7843227

[18] McLean A.R. and Michie C.A., In vivo estimates of division and death rates of human T lymphocytes. Proceedings of the National Academy of Sciences of the United States of America, 1995. 92(9): p. 3707–11. doi: 10.1073/pnas.92.9.3707 7731969 PMC42030

[19] Murray J.M., Zaunders J.J., McBride K.L., Xu Y., Bailey M., Suzuki K., et al., HIV DNA Subspecies Persist in both Activated and Resting Memory CD4+ T Cells during Antiretroviral Therapy. Journal of Virology, 2014. 88(6): p. 3516–3526. doi: 10.1128/JVI.03331-13 24403590 PMC3957951

[20] Lee E., Bacchetti P., Milush J., Shao W., Boritz E., Douek D., et al., Memory CD4 + T-Cells Expressing HLA-DR Contribute to HIV Persistence During Prolonged Antiretroviral Therapy. Frontiers in Microbiology, 2019. 10(2214). doi: 10.3389/fmicb.2019.02214 31611857 PMC6775493

[21] Hill A.L., Rosenbloom D.I.S., Siliciano J.D., and Siliciano R.F., Insufficient Evidence for Rare Activation of Latent HIV in the Absence of Reservoir-Reducing Interventions. PLOS Pathogens, 2016. 12(8): p. e1005679. doi: 10.1371/journal.ppat.1005679 27560936 PMC4999146

[22] Conway J.M. and Perelson A.S., Post-treatment control of HIV infection. Proceedings of the National Academy of Sciences, 2015. 112(17): p. 5467–5472. doi: 10.1073/pnas.1419162112 25870266 PMC4418889

[23] Murray J.M., Latent HIV dynamics and implications for sustained viral suppression in the absence of antiretroviral therapy. J Virus Erad., 2018. 4(2): p. 91–98. 29682300 10.1016/S2055-6640(20)30250-8 PMC5892671

[24] Pankau M.D., Reeves D.B., Harkins E., Ronen K., Jaoko W., Mandaliya K., et al., Dynamics of HIV DNA reservoir seeding in a cohort of superinfected Kenyan women. PLoS Pathog, 2020. 16(2): p. e1008286. doi: 10.1371/journal.ppat.1008286 32023326 PMC7028291

[25] Brodin J., Zanini F., Thebo L., Lanz C., Bratt G., Neher R.A., et al., Establishment and stability of the latent HIV-1 DNA reservoir. Elife, 2016. 5. doi: 10.7554/eLife.18889 27855060 PMC5201419

[26] Abrahams M.R., Joseph S.B., Garrett N., Tyers L., Moeser M., Archin N., et al., The replication-competent HIV-1 latent reservoir is primarily established near the time of therapy initiation. Sci Transl Med, 2019. 11(513). doi: 10.1126/scitranslmed.aaw5589 31597754 PMC7233356

[27] Coffin J.M. and Hughes S.H., Clonal Expansion of Infected CD4+ T Cells in People Living with HIV. Viruses, 2021. 13(10). doi: 10.3390/v13102078 34696507 PMC8537114

[28] Chomont N., El-Far M., Ancuta P., Trautmann L., Procopio F.A., Yassine-Diab B., et al., HIV reservoir size and persistence are driven by T cell survival and homeostatic proliferation. Nature Medicine, 2009. 15(8): p. 893–900. doi: 10.1038/nm.1972 19543283 PMC2859814

[29] Ahmed R. and Gray D., Immunological memory and protective immunity: understanding their relation. Science, 1996. 272(5258): p. 54–60. doi: 10.1126/science.272.5258.54 8600537

[30] Babiker A., Darby S., De Angelis D., Kwart D., Porter K., Beral V., et al., Time from HIV-1 seroconversion to AIDS and death before widespread use of highly-active antiretroviral therapy: a collaborative re-analysis. Collaborative Group on AIDS Incubation and HIV Survival including the CASCADE EU Concerted Action. Concerted Action on SeroConversion to AIDS and Death in Europe. Lancet, 2000. 355(9210): p. 1131–1137. 10791375

[31] Chun T.W., Engel D., Berrey M.M., Shea T., Corey L., and Fauci A.S., Early establishment of a pool of latently infected, resting CD4(+) T cells during primary HIV-1 infection. Proceedings of the National Academy of Sciences of the United States of America, 1998. 95(15): p. 8869–73. doi: 10.1073/pnas.95.15.8869 9671771 PMC21169

[32] Ruff C.T., Ray S.C., Kwon P., Zinn R., Pendleton A., Hutton N., et al., Persistence of Wild-Type Virus and Lack of Temporal Structure in the Latent Reservoir for Human Immunodeficiency Virus Type 1 in Pediatric Patients with Extensive Antiretroviral Exposure. J. Virol., 2002. 76(18): p. 9481–9492. doi: 10.1128/jvi.76.18.9481-9492.2002 12186930 PMC136462

[33] Strain M.C., Gunthard H.F., Havlir D.V., Ignacio C.C., Smith D.M., Leigh-Brown A.J., et al., Heterogeneous clearance rates of long-lived lymphocytes infected with HIV: intrinsic stability predicts lifelong persistence. Proc Natl Acad Sci U S A, 2003. 100(8): p. 4819–24. doi: 10.1073/pnas.0736332100 12684537 PMC153639

[34] Murray J.M., Zaunders J., Emery S., Cooper D.A., Hey-Nguyen W.J., Koelsch K.K., et al., HIV dynamics linked to memory CD4+ T cell homeostasis. PLOS ONE, 2017. 12(10): p. e0186101. doi: 10.1371/journal.pone.0186101 29049331 PMC5648138

[35] Hermankova M., Ray S.C., Ruff C., Powell-Davis M., Ingersoll R., D’Aquila R.T., et al., HIV-1 drug resistance profiles in children and adults with viral load of <50 copies/ml receiving combination therapy. Jama, 2001. 286(2): p. 196–207. doi: 10.1001/jama.286.2.196 11448283

[36] Kieffer T.L., Finucane M.M., Nettles R.E., Quinn T.C., Broman K.W., Ray S.C., et al., Genotypic analysis of HIV-1 drug resistance at the limit of detection: virus production without evolution in treated adults with undetectable HIV loads. J Infect Dis, 2004. 189(8): p. 1452–65. doi: 10.1086/382488 15073683

[37] Ramratnam B., Bonhoeffer S., Binley J., Hurley A., Zhang L., Mittler J.E., et al., Rapid production and clearance of HIV-1 and hepatitis C virus assessed by large volume plasma apheresis. Lancet, 1999. 354(9192): p. 1782–5. doi: 10.1016/S0140-6736(99)02035-8 10577640

[38] De Boer R.J., Ribeiro R.M., and Perelson A.S., Current Estimates for HIV-1 Production Imply Rapid Viral Clearance in Lymphoid Tissues. PLOS Computational Biology, 2010. 6(9): p. e1000906. doi: 10.1371/journal.pcbi.1000906 20824126 PMC2932679

[39] Hill A.L., Rosenbloom D.I.S., Fu F., Nowak M.A., and Siliciano R.F., Predicting the outcomes of treatment to eradicate the latent reservoir for HIV-1. Proceedings of the National Academy of Sciences, 2014. 111(37): p. 13475–13480. doi: 10.1073/pnas.1406663111 25097264 PMC4169952

[40] Chun T.W., Carruth L., Finzi D., Shen X., DiGiuseppe J.A., Taylor H., et al., Quantification of latent tissue reservoirs and total body viral load in HIV-1 infection. Nature, 1997. 387(6629): p. 183–8. doi: 10.1038/387183a0 9144289

[41] Siliciano J.D., Kajdas J., Finzi D., Quinn T.C., Chadwick K., Margolick J.B., et al., Long-term follow-up studies confirm the stability of the latent reservoir for HIV-1 in resting CD4+ T cells. Nat Med, 2003. 9(6): p. 727–8. doi: 10.1038/nm880 12754504

[42] Peluso M.J., Bacchetti P., Ritter K.D., Beg S., Lai J., Martin J.N., et al., Differential decay of intact and defective proviral DNA in HIV-1-infected individuals on suppressive antiretroviral therapy. JCI Insight, 2020. 5(4). doi: 10.1172/jci.insight.132997 32045386 PMC7101154

[43] Gaebler C., Falcinelli S.D., Stoffel E., Read J., Murtagh R., Oliveira T.Y., et al., Sequence Evaluation and Comparative Analysis of Novel Assays for Intact Proviral HIV-1 DNA. J Virol, 2021. 95(6). doi: 10.1128/JVI.01986-20 33361426 PMC8094944

[44] Bruner K.M., Murray A.J., Pollack R.A., Soliman M.G., Laskey S.B., Capoferri A.A., et al., Defective proviruses rapidly accumulate during acute HIV-1 infection. Nat Med, 2016. 22(9): p. 1043–9. doi: 10.1038/nm.4156 27500724 PMC5014606

[45] Ho Y.-C., Shan L., Nina N., Hosmane, Wang J., Laskey Sarah B., Rosenbloom Daniel I.S., et al., Replication-Competent Noninduced Proviruses in the Latent Reservoir Increase Barrier to HIV-1 Cure. Cell, 2013. 155(3): p. 540–551. doi: 10.1016/j.cell.2013.09.020 24243014 PMC3896327

[46] Palmer S., Maldarelli F., Wiegand A., Bernstein B., Hanna G.J., Brun S.C., et al., Low-level viremia persists for at least 7 years in patients on suppressive antiretroviral therapy. Proceedings of the National Academy of Sciences of the United States of America, 2008. 105(10): p. 3879–84. doi: 10.1073/pnas.0800050105 18332425 PMC2268833

