# Supplementary material for: Dynamics of latent HIV under clonal expansion
Source: PLoS Pathog. 2021 Dec 20;17(12):e1010165. doi: 10.1371/journal.ppat.1010165 (PMC8722732; doi:10.1371/journal.ppat.1010165)
Supplement: S1 Fig — (DOCX) [file ppat.1010165.s001.docx]

### S1 Fig: Latent reservoir dynamics prior to ART


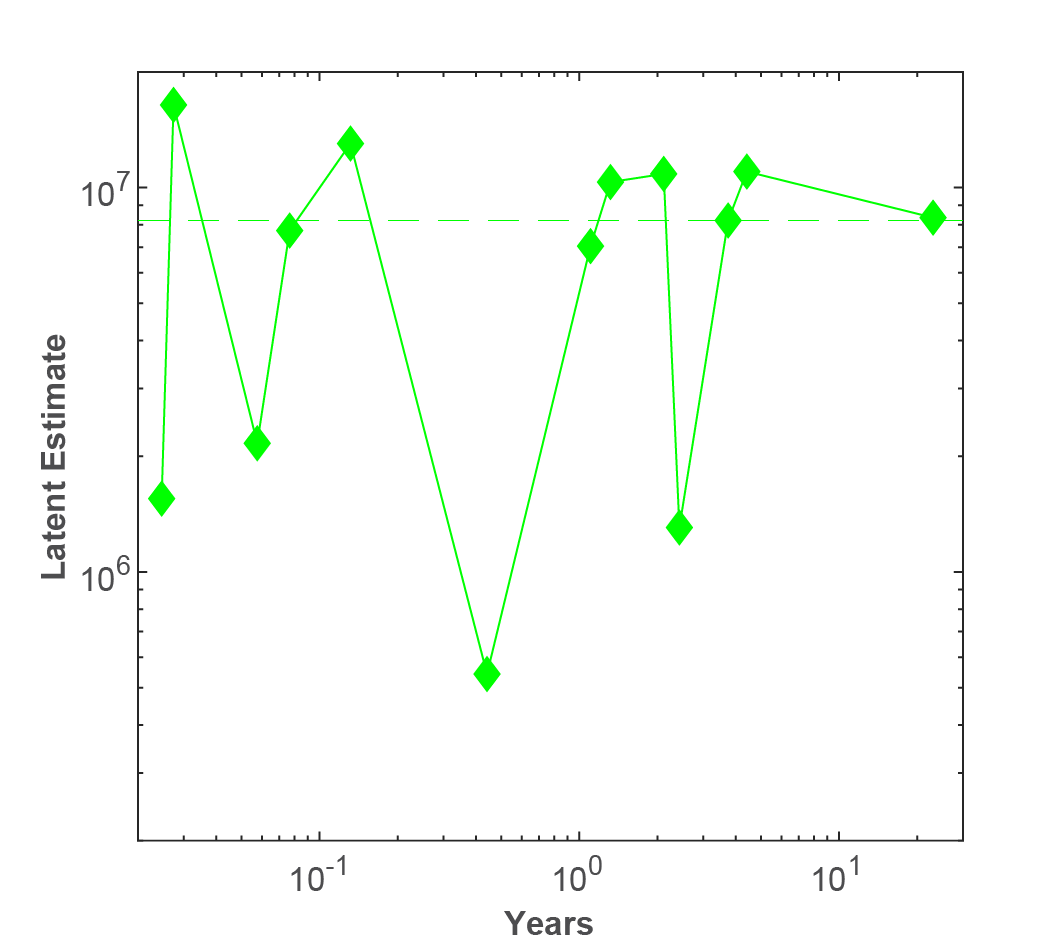


S1 Fig: estimated total integrated HIV DNA against duration of infection prior to ART showing relatively stable levels after initial infection (median value of $8.2\times{10}^{6}$ cells shown as dashed line). These values were obtained by modelling second phase integrated HIV DNA decay rates based on data using a nested real-time PCR assay (1).

References

1. Murray JM, McBride K, Boesecke C, Bailey M, Amin J, Suzuki K, et al. Integrated HIV DNA accumulates prior to treatment while episomal HIV DNA records ongoing transmission afterwards. AIDS. 2012;26(5):543-50.
