# Supplementary material for: Dynamics of latent HIV under clonal expansion
Source: PLoS Pathog. 2021 Dec 20;17(12):e1010165. doi: 10.1371/journal.ppat.1010165 (PMC8722732; doi:10.1371/journal.ppat.1010165)
Supplement: S2 Fig — (DOCX) [file ppat.1010165.s002.docx]

### S2 Fig: Changes in the proliferative profile and the times of establishment of each clone in the first individual simulation.


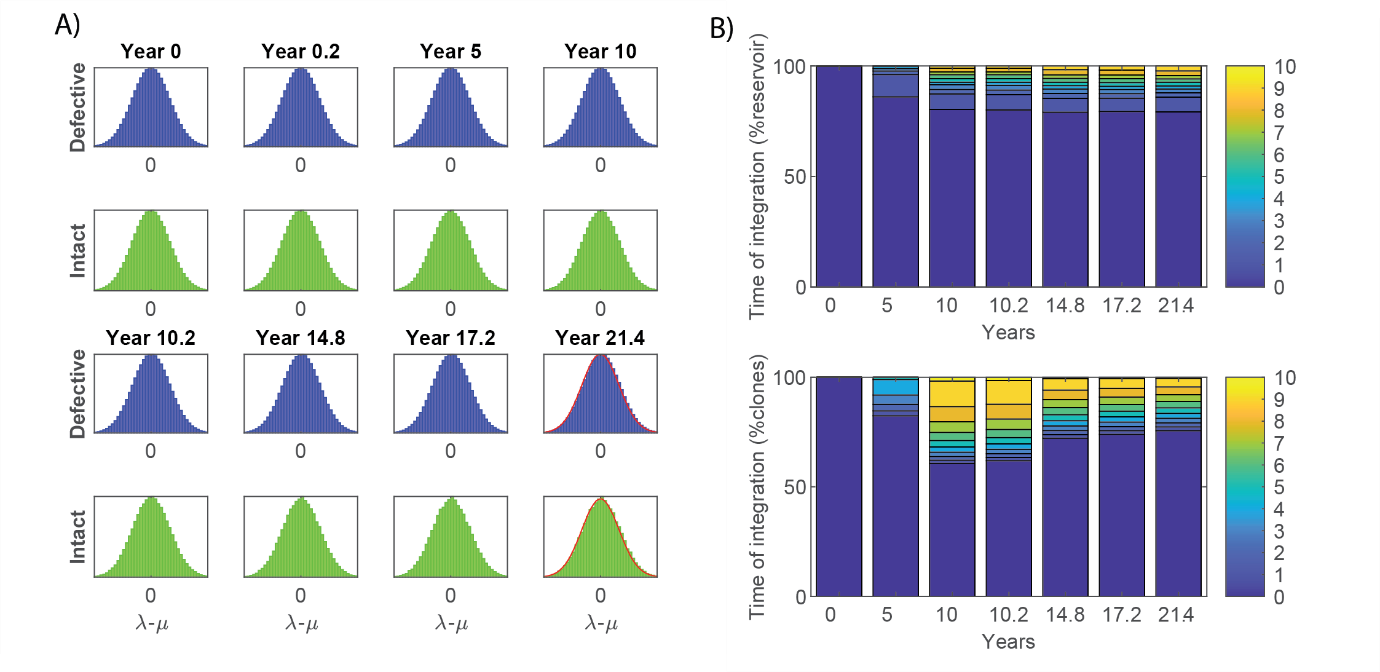


S2 Fig: A) The distribution of the reservoir gradually shifts to higher proliferative levels as determined by the surviving proportion of clonal populations having higher values of $\lambda$ relative to death $\mu$. In the last year the initial proliferative distribution is shown as a red line for comparison. B) Percentages of total number of latent cells (% reservoir) and distinct clonal populations (%clones) surviving at each time categorized by the times of their establishment (when that clone was first seeded into the reservoir). Since no new infections are assumed to occur during ART, integration times range up to ART initiation at year 10. At year 11.4 of ART, 2.4% of the reservoir was established within the year before ART initiation, compared to 79% for the first year of infection.
