# Supplementary material for: Dynamics of latent HIV under clonal expansion
Source: PLoS Pathog. 2021 Dec 20;17(12):e1010165. doi: 10.1371/journal.ppat.1010165 (PMC8722732; doi:10.1371/journal.ppat.1010165)
Supplement: S3 Fig — (DOCX) [file ppat.1010165.s003.docx]

### S3 Fig: Histograms of clonal sizes for the child simulation.


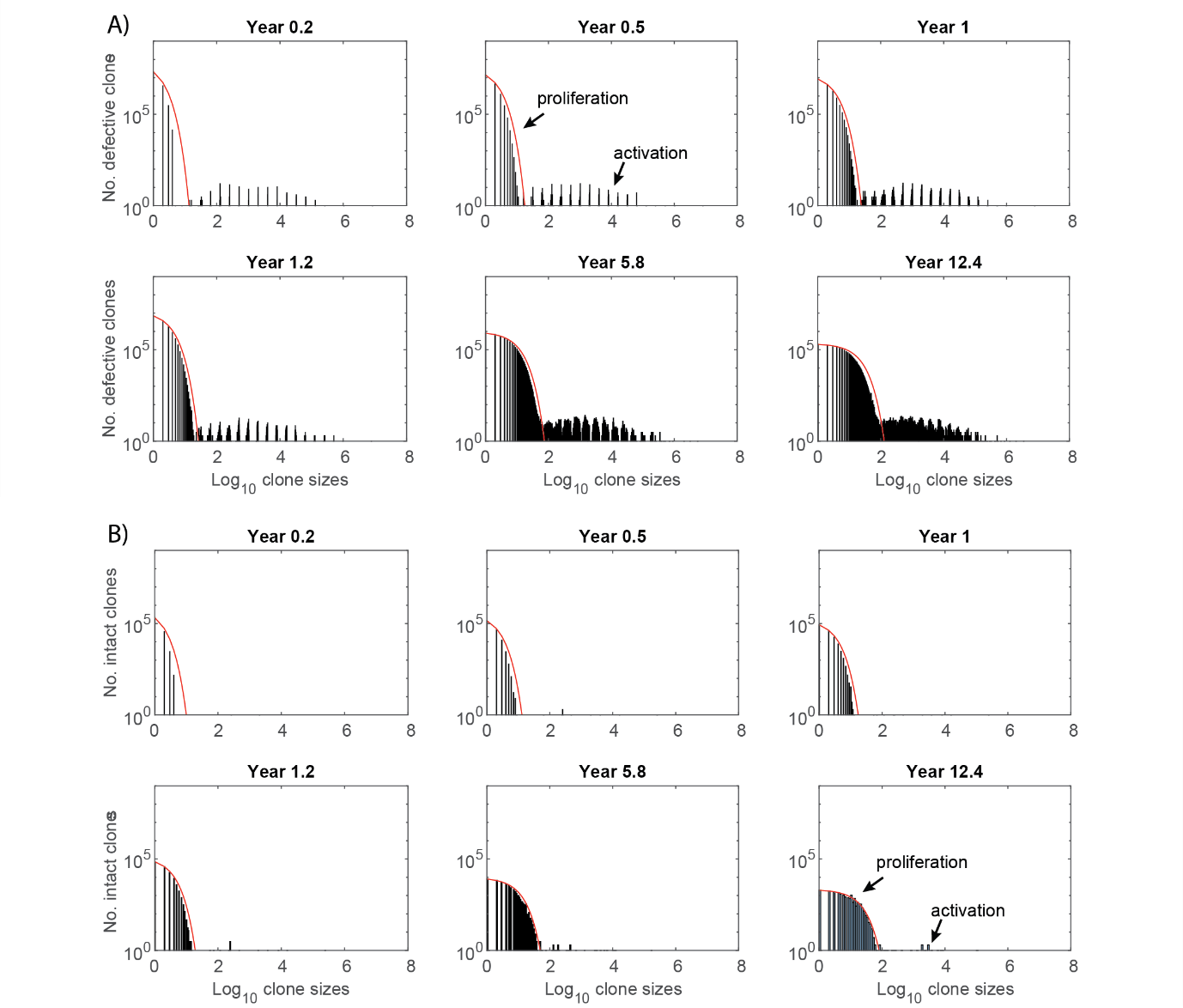


S3 Fig: Histograms of A) defective and B) intact clone sizes. First row in each plot shows times prior to ART, while second row shows times during ART. The simulations were conducted as for the adult calculation but with a one year period prior to ART, and a 10-fold lower initial seeding of the latent reservoir. Parameters were as described in Fig 8.
