# Supplementary material for: Dynamics of latent HIV under clonal expansion
Source: PLoS Pathog. 2021 Dec 20;17(12):e1010165. doi: 10.1371/journal.ppat.1010165 (PMC8722732; doi:10.1371/journal.ppat.1010165)
Supplement: S4 Fig — (DOCX) [file ppat.1010165.s004.docx]

### S4 Fig: Simulations with higher levels of reseeding.


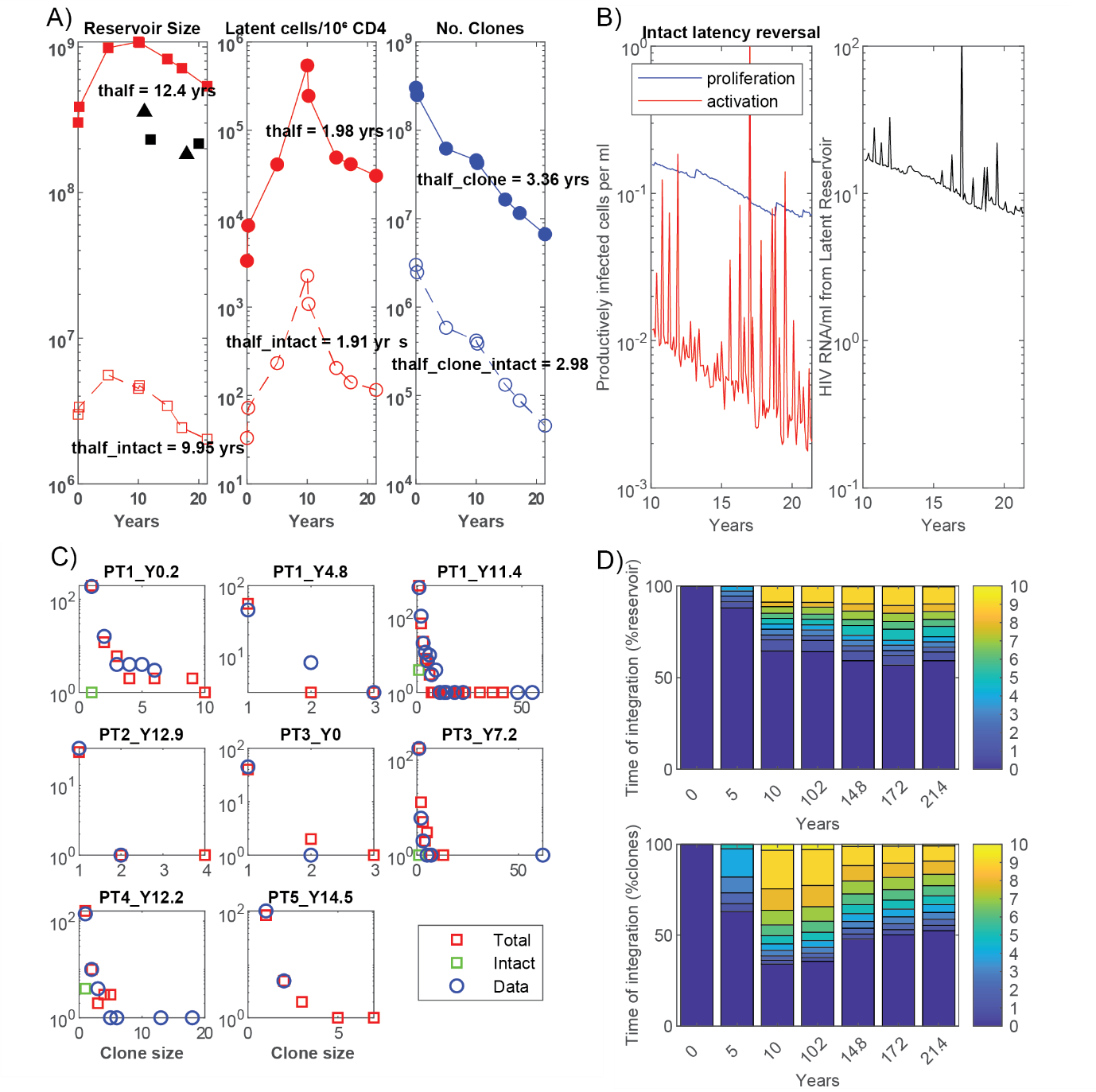


S4 Fig: Simulation with higher levels of reseeding. The parameter set that was second best in fitting the Patient 1 Year 11.4 clonal distribution had higher levels of reseeding throughout the course of untreated infection ($\bar{s}$=20 so that the annual level of reseeding was 1/20^th^ the amount at PHI, compared to $\bar{s}$=42). Parameters: µ=1.0076, λ_v_=1.86×10^-4^, α=0.0063, p_α_=0.985, p_λ_=0.965, n_div_=10, $\bar{s}$=20, m_act_=7. The mean cell life-span is 1/µ=0.992 years or 11.9 months. At year 11.4 of ART, 9.7% of the reservoir was established within the year before ART initiation, compared to 59% for the first year of infection.
