# Supplementary material for: Dynamics of latent HIV under clonal expansion
Source: PLoS Pathog. 2021 Dec 20;17(12):e1010165. doi: 10.1371/journal.ppat.1010165 (PMC8722732; doi:10.1371/journal.ppat.1010165)
Supplement: S1 Table — (DOCX) [file ppat.1010165.s005.docx]

### S1 Table: Sensitivity analysis

Correlation (p and r values) for outputs in terms of the model parameters based on the full 2,000 LHS parameter sets and calculated only via the intact reservoir component. The outputs are the rate of reservoir change prior to ART, the rate of reservoir change during ART, ${log}_{10} HIV DNA$ copies at the start of ART, the linear rate of change of pVL during ART.

| p values | Res. rate preART | Res. rate ART | $\log_{10} pVL(0)$ | $pVL rate$ |
| --- | --- | --- | --- | --- |
| $\mu$ | **4.68E-88** | **4.73E-191** | 3.16E-07 | 3.42E-01 |
| $\lambda_{v}$ | 4.06E-32 | 5.79E-74 | 1.24E-04 | 6.46E-01 |
| $\alpha$ | 1.68E-35 | 7.38E-65 | 1.16E-31 | 1.63E-04 |
| $p_{\alpha}$ | 5.08E-05 | 6.67E-07 | 2.50E-52 | 2.37E-04 |
| $p_{\lambda}$ | 6.43E-01 | 2.82E-01 | 5.94E-21 | 9.97E-02 |
| $n_{div}$ | 1.97E-10 | 6.17E-06 | **1.77E-114** | **6.23E-11** |
| $\bar{s}$ | 2.84E-12 | 3.96E-02 | 7.95E-06 | 2.65E-01 |
| $m_{act}$ | 1.26E-01 | 9.78E-02 | 5.56E-01 | 1.26E-02 |

| r values | Res. rate preART | Res. rate ART | $\log_{10} pVL(0)$ | $pVL rate$ |
| --- | --- | --- | --- | --- |
| $\mu$ | **-0.427** | **-0.598** | -0.115 | -0.021 |
| $\lambda_{v}$ | -0.262 | -0.394 | -0.086 | -0.010 |
| $\alpha$ | -0.275 | -0.370 | 0.260 | -0.085 |
| $p_{\alpha}$ | -0.091 | -0.112 | -0.334 | 0.083 |
| $p_{\lambda}$ | 0.010 | 0.024 | -0.210 | 0.037 |
| $n_{div}$ | 0.143 | 0.102 | **0.481** | **-0.147** |
| $\bar{s}$ | -0.157 | -0.046 | -0.101 | 0.025 |
| $m_{act}$ | -0.035 | -0.037 | 0.013 | -0.056 |
