# Supplementary material for: Dynamics of latent HIV under clonal expansion
Source: PLoS Pathog. 2021 Dec 20;17(12):e1010165. doi: 10.1371/journal.ppat.1010165 (PMC8722732; doi:10.1371/journal.ppat.1010165)
Supplement: S1 Text — (DOCX) [file ppat.1010165.s006.docx]

### S1 Text: Clonal expansion under proliferation

A simple model of expanding clone sizes due to proliferation, with loss from death and activation, can be described by the following model where $D_{i}$ represents the number of latently infected clones of size $i$

$$\frac{dD_{1}}{dt}=\gamma A-\lambda D_{1}-\mu D_{1}+2\mu D_{2}-\alpha D_{1}$$

$$\frac{dD_{i}}{dt}=\left( i-1 \right)\lambda D_{i-1}-i\lambda D_{i}-i\mu D_{i}+\left( i+1 \right)\mu D_{i+1}-\alpha D_{i}, i=2, \ldots$$

The first term represents establishment of latently infected singletons at rate $\gamma$ from productively infected cells $A$. Death of a cell within a clone of size $i+1$ at a per cell rate of $\mu$ reduces that clone to size $i$ (increasing the value of $D_{i}$ by 1), while proliferation at a per cell rate of $\lambda$ reverses that process. In this model all clones exhibit the same value of $\lambda$. Here activation of the clone at rate $\alpha$ removes that clone completely (since this model only investigates the component of the latent reservoir that is expanded through proliferation). In this model no differences are assumed for defective or intact virus, otherwise separate components can be described with these same equations. The total number of cells in the reservoir is given by

$$D=\sum_{i=1} iD_{i}$$

Combining this expression with the differential equations, and after simplification, gives the simpler equation for total reservoir dynamics

$$\frac{dD}{dt}=\gamma A+\left( \lambda-\alpha-\mu\right)D$$

which decays at rate $\lambda-\alpha-\mu$ assuming little replenishment from residual viremia during ART. The total number of clones $\Delta=\sum_{i} D_{i}$ follows a slightly different dynamic

$$\frac{d\Delta}{dt}=\gamma A-\mu D_{1}-\alpha\Delta$$

Numerical solutions of the total reservoir model show $D_{i}$ decaying roughly exponentially with clone size $i$ so that $D_{i}\approx\bar{D} z^{i-1}$. In that case for $i\geq2$

$$\frac{dD_{i}}{dt}=\left( i-1 \right)\lambda D_{i-1}-i\lambda D_{i}-i\mu D_{i}+\left( i+1 \right)\mu D_{i+1}-\alpha D_{i}$$

$$=\bar{D}z^{i-2}\left[ \left( i-1 \right)\lambda-i\left( \lambda+\mu\right)z+\left( i+1 \right)\mu z^{2}-\alpha z \right]$$

By definition we also have

$$\frac{dD_{i}}{dt}=\frac{d}{dt}\bar{D}z^{i-1}=z^{i-1}\frac{d}{dt}\bar{D}+\bar{D}\left( i-1 \right)z^{i-2}\frac{dz}{dt}$$

$$=\bar{D}z^{i-2}\left[ \frac{z}{\bar{D}} \frac{d}{dt}\bar{D}+\left( i-1 \right)\frac{dz}{dt} \right]$$

Comparing components of $i$ gives

$$\frac{dz}{dt}=\mu\left( z-1 \right)\left( z-\frac{\lambda}{\mu} \right)$$

Since all clones start at PHI at size 1, $z$ is initially small and asymptotes to $\frac{\lambda}{\mu}$ if this is smaller than 1, otherwise the series diverges, with clones and the reservoir eventually growing exponentially in size. Comparisons with the second individual simulation of these estimates of clone size $\bar{D} z^{i-1}(t)$ where $\bar{D}$ is the number of singletons at the calculated times are shown in Fig 11.

The total number of clones driven solely by proliferation or initial infection is given by

$$\Delta=\sum_{i} D_{i}=\bar{D}\sum_{i=1} z^{i-1}\approx\frac{\bar{D}}{1-z}$$

In that case the size of the reservoir from these proliferating clones is given by

$$D=\sum_{i} iD_{i}=\bar{D}\sum_{i=1} {iz}^{i-1}=\frac{\partial\Delta}{\partial z}=\frac{\bar{D}}{\left( 1-z \right)^{2}}=\frac{\Delta^{2}}{\bar{D}}$$

and their mean clone size is given by the ratio

$$Mean clone size=\frac{\sum_{i} iD_{i}}{\sum_{i} D_{i}}=\frac{D}{\Delta}\approx\frac{1}{1-z}$$

With little or no new infection during ART, mean clone size will increase since

$$\frac{d}{dt}\left( Mean clone size \right)\approx\frac{1}{\left( 1-z \right)^{2}}\frac{dz}{dt}>0$$

Using the calculation above, the mean size for clones arising from proliferation is 17.5. The mean size of all clones in the simulation described in Fig 11 is much higher at 46.7 since it will include the very high sizes arising from activation as well. On the other hand, the median clone size from that simulation is only 7 since it is mostly determined from the larger number of clones that have been expanded by proliferation (8,315,740 of size 232 or less) than activation (19,160).
